# Impact of socioeconomic factors on pediatric atopic dermatitis population

**DOI:** 10.13105/wjma.v13.i2.105511

**Published:** 2025-06-18

**Authors:** Srilakshmi Haripriya Ponukumati, Rahul Mittal, Barbara Ann Tafuto

**Affiliations:** Department of Health Informatics, Rutgers School of Health Professions, Newark, NJ 07107, United States

**Keywords:** Atopic dermatitis, Socioeconomic factors, Eczema, Neurodermatitis, Disseminated neurodermatitis, Infantile eczema, Socioeconomic disparities

## Abstract

**BACKGROUND:**

Atopic dermatitis (AD), or eczema, is a chronic, pruritic inflammatory skin disease affecting children and adults. Socioeconomic status (SES) plays a significant role in developing AD. However, mixed evidence from a previous study by Bajwa *et al* makes it difficult to determine the directionality of the association. There is a literature gap in understanding the causal association between AD and socioeconomic factors.

**AIM:**

To evaluate the impact of disparities in SES on pediatric AD populations.

**METHODS:**

Based on the eligibility criteria, the literature review identified eight articles since July 2021, and a descriptive analysis was conducted using an Excel spreadsheet on key components collected from the identified studies.

**RESULTS:**

Eight observational studies assessed SES in pediatric AD. Five observational studies showed mixed associations between AD and SES. Sub-analysis revealed that urban areas had a higher prevalence of AD, and four studies identified a positive association between parental education and AD in the pediatric population. Socioeconomic variables, such as residential areas and household income, significantly influence disease outcomes.

**CONCLUSION:**

There is mixed association between pediatric AD and SES, with AD positively associated with parental education. There is critical need to evaluate global impact of SES variables on pediatric AD.

## INTRODUCTION

Atopic dermatitis (AD), or eczema, is a chronic, pruritic inflammatory skin disease that typically begins in childhood but can affect individuals at any age. Multiple factors play a significant role in AD development, including genetics, environment, and the immune system, and these may also contribute to the exacerbation or remission of AD. Symptoms of AD include swelling, redness, cracking, crusting, scaling, and leakage of clear fluid in extreme conditions. Complications from AD can include bacterial and viral skin infections, sleep loss, conjunctivitis, and hand eczema. While interventional therapy can help control symptoms based on their location, severity, and potential flare-ups, the permanent treatment and/or cure of AD has proven challenging[1].

A survey on AD prevalence among pediatric populations aged 6 months to < 18 years across 18 countries showed that a significant percentage of the population is affected by AD. However, less than 15% of this patient group had severe AD [2]. A recent 2023 epidemiological survey highlighted that 41.5% of countries lack data on AD, even though 102.78 million pediatric individuals are affected globally. AD epidemiology varies by age and sex across various geographical regions [3]. For example, in developed countries, approximately 10%-30% of pediatric patients have AD. Of those, 60% are diagnosed with early-onset AD within the 1^st^ year of age, and it then resolves by 12 years of age[4]. Despite the high prevalence of AD, there is a significant gap in our understanding of the global disease distribution.

Recent research highlights the significant global burden of AD, with a higher prevalence in young children, females, and pediatric population in resource-rich countries[5]. In Asia, rising AD rates are largely associated with socioeconomic and environmental factors, including lifestyle, parental education, family income, and metropolitan living[6]. In low-resource settings like India, these factors shape ethnic treatment choices, underscoring the need for greater disease awareness, nutrition, and parental counseling to manage disease severity and complications[7]. Enhancing disease-specific awareness in these regions may also improve child health outcomes, particularly among parents with limited health literacy[8]. Conversely, in developed countries like the United States, AD incidence is higher in children from single-parent households, those with unmarried mothers, or those living with nonbiologic fathers, often leading to poorer physical and mental health outcomes in affected children[9]. Given these insights, further exploration of AD’s socioeconomic impact on pediatric populations is crucial.

Bajwa *et al*[10] conducted a systematic review examining the relationship between AD and socioeconomic position, highlighting mixed findings across studies. These inconsistencies underscore the need for further research to assess how socioeconomic factors influence disease severity and management in pediatric AD populations. To address this gap, the current review explores the impact of socioeconomic status (SES) on pediatric AD, particularly its effects on disease progression and complications.

## MATERIALS AND METHODS

This literature review explores the following question: How does SES (E) affect the pediatric AD population (P) in ways that contribute to disease-specific complications (O)? The PRISMA Statement (Preferred Reporting Items for Systematic Review and Meta-Analyses) was used as a framework to ensure a structured and systematic approach to this literature review[11].

### Eligibility criteria

#### Inclusion criteria:

(1) Articles examining the relationship between AD and SES; (2) Comparative, case-control, or cohort studies; (3) Participants aged birth to 18 years, including both males and females; (4) Studies published after July 4, 2021; (5) Articles in any language; and (6) Studies that provide separate data for adult and pediatric populations.

#### Exclusion criteria:

(1) Articles not focused on AD and SES; (2) Review articles, commentaries, or studies comparing global data across countries; (3) Studies including only patients over 18 years of age; (4) Articles published before July 4, 2021; and (5) Studies that did not separate pediatric and adult population data.

### Search strategy and study selection

A comprehensive literature search was conducted using keywords and MeSH terms in PubMed, MEDLINE, CINAHL, Embase, and Cochrane databases. Keywords related to AD and alternative terms for SES were identified through the PubMed database and the National Library of Medicine MeSH browser. The following search syntax was applied to retrieve relevant articles published up to October 6, 2024.

#### Search strategy syntax:

(1) AD terms: “Atopic Dermatitis” OR “Eczema, Atopic” OR “Atopic Eczema” OR “Neurodermatitis, Atopic” OR “Atopic Neurodermatitis” OR “Neurodermatitis, Disseminated” OR “Disseminated Neurodermatitis” OR “Eczema, Infantile” OR “Infantile Eczema”; (2) SES terms: “Factor, Socioeconomic” OR “Socioeconomic Factor” OR “Social and Economic Factors” OR “Economic and Social Factors” OR “Socioeconomic Characteristics” OR “Characteristic, Socioeconomic” OR “Socioeconomic Characteristic” OR “Factors, Socioeconomic” OR “High-Income Population” OR “High Income Population” OR “High-Income Populations” OR “Population, High-Income” OR “Land Tenure” OR “Tenure, Land” OR “Standard of Living” OR “Living Standard” OR “Living Standards” OR “Social Inequality” OR “Inequality, Social” OR “Social Inequalities”; and (3) Final search query: Search #1 AND Search #2.

### Data collection

Key study components were extracted and organized in an Excel spreadsheet, which included (1) Study summary characteristics (Table 1): Author, publication year, country, geographical zone, study type, sample size, patient population, age group, sex distribution, exposure, and primary outcome measurement; (2) Study exposure components (Table 2): SES variables including occupation, household income, housing characteristics, parental or participant education, residential area/geographical location, socioeconomic classification, and health insurance; and (3) Study outcome measures (Tables 3 and 4): Associations between AD and SES, AD and parental education, and AD and residential location (urban, rural, and other classification type).

Since all included studies were observational, the Critical Appraisal Skills Program (CASP) 2024 checklist for cohort studies[12] was used to assess bias (Table 5). Bias ratings were determined based on the responses of "yes," "can’t say," and "no" for each dimension of the study.

### Outcome measures

The primary objective of the literature review was to assess the impact of SES on pediatric AD patients. Key outcome measures included: SES, residential area/geographical location, parental or participant education, household income, and health insurance. These factors were analyzed to determine their influence on disease outcomes in pediatric AD populations.

### Statistical analysis

This review assessed the identified studies using a descriptive statistical analysis approach without incorporating meta-analysis.

## RESULTS

**Figure 1:**
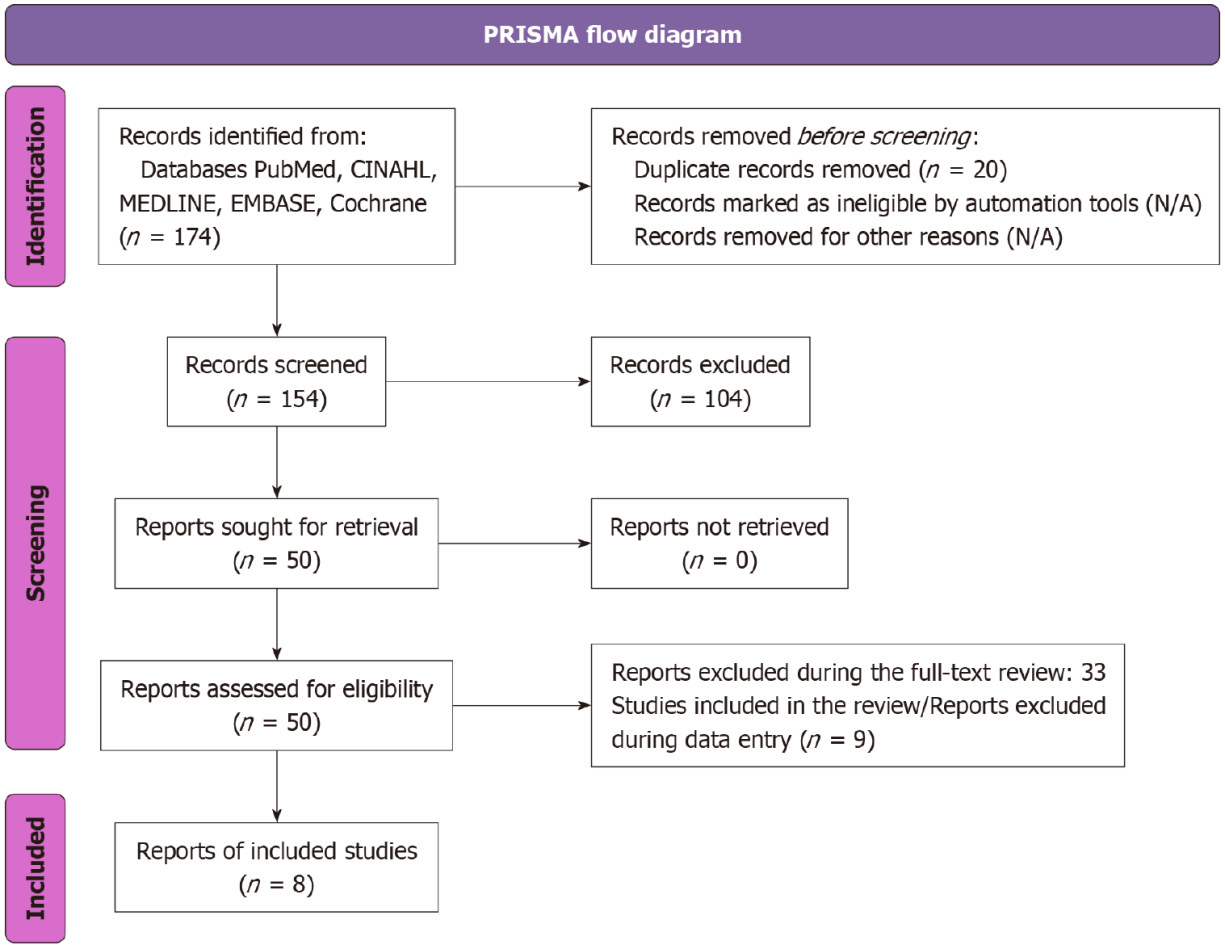
PRISMA flow diagram[13].

The literature search initially identified 1179 articles, of which 174 were published since July 2021. A total of 104 articles were excluded based on author names, titles, and abstracts, while 20 duplicate articles were excluded after applying inclusion and exclusion criteria. From the remaining 50 articles, 33 were excluded during the full-text review, and 9 were excluded during data entry. The final set of eight articles underwent a thorough pearl-growing search to identify additional studies. However, no further articles were identified through this process. These eight articles were finalized for the descriptive research analysis[14-21]. Figure 1 provides the PRISMA flow diagram of the search process[13].

### Study summary characteristics

Among the eight included studies, 37.5% were conducted in Asia, 25% in the Americas, 25% in the Middle East, and 12.5% in Europe[14-21]. The studies included five cross-sectional studies, one survey, one prospective descriptive study, and one retrospective case-control study, all of which assessed SES in the AD population[14-21]. Regarding sample size, five studies had fewer than 1000 participants, one study had 2048 participants, and two studies included more than 75000 participants[14-21]. The age range varied from birth to 18 years in seven studies, while one study included participants ranging from 2 months to 30 years, with separate data collected for pediatric and young populations[14-21]. Demographically, seven studies compared male and female participants[14-21] (Table 1).

### Socioeconomic variables

The eight studies included in the review evaluated various socioeconomic factors, including occupation, household income, housing characteristics, parental or participant education, residential area/geographical location, SES, and health insurance[14-21] (Table 2).

### Outcomes

Seven studies examined the relationship between AD and SES within the study population. Of these, five found a mixed association, one identified a positive association, and one found no association[14-21] (Table 3).

### Sub-analysis

#### Residential area:

Four studies included data on participants' residential areas, categorized as urban or rural, and one study distinguished between apartment/attached house and house. A sub-analysis of the urban *vs* rural data revealed a higher prevalence of AD in urban areas compared to rural areas[14,16,18-20] (Table 4).

#### Parental or participant education:

Two studies analyzed maternal and paternal education as variables and found a positive association between AD and SES. Additionally, two studies focused on parental education as a variable and concluded a positive association with SES[14-21] (Table 3).

#### Risk of bias:

The CASP checklist for cross-sectional studies was used to assess the risk of bias in the observational studies. The risk of bias assessment revealed that one study had a low risk of bias, two studies had a medium risk of bias, and five studies had a high risk of bias[14-21] (Table 5).

## DISCUSSION

Bajwa *et al*[10] performed a systematic review focusing on the pediatric population, finding mixed evidence regarding the association between AD and SES, with 45.7% showing a positive association, 13.3% showing a negative association, and 41% showing no association. In contrast, this literature review found similarly mixed evidence in the association between AD and SES in pediatric populations[14-21]. However, the majority (71.4%) of studies reported mixed associations, with limited evidence for a positive association. This limited evidence highlights differences in the directionality of the association when compared to the findings of the Bajwa *et al*[10] systematic review.

Socioeconomic factors play a significant role in shaping individual health outcomes and contributing to public health challenges. The prevalence and burden of AD are influenced by environmental factors, with SES and privileged circumstances serving as key risk factors that affect the expression of the disease[22]. In the Bajwa *et al*[10] review, a large proportion of pediatric studies assessed the evidence of socioeconomic position based on parental education (41.9%) and income (37%), followed by class/occupation (14.28%). In contrast, this review measured the association between AD and SES based on a broader definition of SES (87.5%)[14-21]. This variation underscores the fact that individual studies define and measure socioeconomic position according to the country-specific definition of SES employed in each study.

Socioeconomic disparities have a significant impact on healthcare access and the quality of therapeutic management, exacerbating existing challenges[22]. In this literature review, 62.5% of studies identified residential areas as a key socioeconomic determinant in evaluating the association between AD and SES among pediatric populations[14,16,18-20]. This indicates substantial evidence that researchers have analyzed these data to better understand the complexities of the association between AD and SES. Notably, 50% of the studies reported a significant proportion of participants residing in urban areas. In contrast, rural populations demonstrated limited healthcare utilization, which contributed to a reduced quality of life in the pediatric AD population. These findings are consistent with the systematic review by Shin *et al*[23], which suggested that lower prevalence of AD in rural areas may be attributed to limited disease awareness, lower education levels, and restricted access to medical care compared to urban areas.

Parental education, particularly maternal education, plays a crucial role in shaping lifestyle choices, healthy behaviors, and the development of atopic disorders[24,25]. Limited parental health literacy can reduce the effectiveness of treatment in children with chronic diseases, while adequate literacy improves parental involvement and satisfaction with care[26]. Similarly, in the Bajwa *et al*[10] study, this literature review found that parental or participant education accounted for 50% of the socioeconomic variables used to assess the significant correlation between the prevalence of AD and SES[14,17-19]. However, previous systematic review did not make specific recommendations regarding parental education[10]. Our review highlights the significance of parental education in children with AD. Additionally, the findings suggest that underrepresented minority groups in high-income countries face linguistic and cultural barriers, which limit their access to healthcare and negatively impact on their quality of life[17]. These observations align with the findings of a narrative review that identified cultural and linguistic barriers as major challenges to healthcare access[27]. The considerable amount of missing data across studies underscores the need for more comprehensive and standardized reporting in future research.

Household income is another key socioeconomic factor that can significantly influence AD severity. A study by Chung *et al*[28] on the socioeconomics of AD identified a positive correlation between higher SES and AD prevalence, while lower SES was associated with increased AD severity. In addition to the significant direct healthcare costs, indirect costs, such as lost educational opportunities and missed school attendance, further contribute to the AD burden. Similarly, a prospective birth cohort study of Japanese participants identified low household income as a risk factor for physician-diagnosed eczema and asthma, highlighting the importance of physicians recognizing low-income families as vulnerable populations[29]. Compared to the systematic review by Bajwa *et al*[10], this study reveals a growing interest among researchers in exploring the association between household income and AD in pediatric populations[14-15,17,19].

SES plays a significant role in determining access to healthcare. Inequalities in healthcare remains a persistent challenge; however, improving healthcare quality can substantially reduce disease burden and enhance patient care[30]. In countries like India, AD poses a considerable burden, exacerbated by the limited availability of trained professionals in allergy medicine and an inadequate healthcare infrastructure[31]. In their systematic review, De *et al*[32] identified medication costs as a major contributor to the economic burden of AD, emphasizing the lack of comprehensive epidemiological data across the country. Globally, there is an increasing urgency to improve healthcare access and quality. Compared to the Bajwa *et al*[10] systematic review, this literature review highlights a significant shift to including healthcare insurance as a socioeconomic variable[14].

The 2023 clinical practice guidelines[33] propose an eczema action plan, an educational intervention designed to support parents and caregivers in self-managing AD and improving disease control. Randomized controlled trials have been conducted to assess the effectiveness of this intervention, which is highly valued by physicians, patients, and caregivers. However, addressing the socioeconomic burden of AD in pediatric populations remains a significant challenge, especially given the global variability in socioeconomic factors. Though 2021 Bajwa *et al*’s study[10] determined the significance of socioeconomic factors in AD, 2023 clinical practice guidelines did not specify any recommendations on socioeconomic factors and its impact on overall AD burden. Policy makers must address the significance of socioeconomic factors and their influence on AD and develop strategies to reduce the overall burden to improve outcomes for children with AD.

This literature review has several limitations. First, the measurement of SES varies significantly across studies due to differences in regional definitions and socioeconomic indicators, which complicates the comparison of variables and the generalization of the findings. Given the geographical differences in socioeconomic factors due to population-level variables, developing region-specific strategies targeting potential factors could help reduce the AD burden in the pediatric population[34]. Second, reliance on cross-sectional studies limits the ability to establish causal relationships between SES and AD. Despite the limitations of cross-sectional studies in establishing causality, over 50% of the studies reviewed were based on large population sample size greater than 500 participants, enhancing the reliability and generalizability of their findings. Third, the limited number of studies published since the last systematic review results in insufficient evidence to draw definitive conclusions about the association between AD and SES. However, this review, conducted from July 4, 2021 to October 6, 2024, identified eight recent observational studies, serving as an adjunct to the systematic review by Bajwa *et al*[10], which included 68 studies focused solely on pediatric populations over more than four decades. The fourth limitation is that various risk factors, longitudinal study design, and health policies act as confounders when evaluating the association between socioeconomic factors and AD. The previous systematic review by Bajwa *et al*[10] did specify confounding factors. However, the study did not adjust the confounders or used the study design to consider confounding factors while analyzing the data. In this review, most of the studies discussed and adjusted the confounders impacting AD thereby increasing the reliability of study results. Future studies should adjust for confounders to better understand the relationship between SES and AD, ideally using longitudinal study design. Lastly, most studies are region-specific or country-specific, and the lack of multicenter studies may introduces regional bias, limits global applicability, and further restricts the ability to collect comprehensive SES data across diverse populations with AD. Conducting multicenter studies may help overcome the limitation by enabling broader SES data collection and improving the global generalizability of the results.

## CONCLUSION

This literature review underscores the multifaceted and dynamic relationship between AD and SES in pediatric populations. Although existing studies present mixed findings, a consistent positive correlation emerges between higher parental education levels and increased prevalence or diagnosis of pediatric AD. Broader socioeconomic determinants—such as geographic residence, household income, and access to healthcare—exert a significant influence on disease severity, management, and quality of life outcomes. The cumulative evidence from this review highlights persistent socioeconomic disparities that disproportionately affect vulnerable and underrepresented pediatric populations.

To address these disparities, future research should adopt multicenter, longitudinal designs with standardized methodologies that account for regional SES variability. Such approaches are essential to disentangle causative mechanisms, inform equitable healthcare strategies, and guide policy development aimed at mitigating SES-related inequities in pediatric AD care and outcomes.

## Figures and Tables

**Table 1:** Study summary characteristics[14-21]

Ref.	Country	Geographical zone	Study type	Sample size	Patient population	Age group	Males, %	Females, %	Exposure	Primary outcome measurement
Reimer-Taschenbrecker *et al*[14], 2023	Chicago, United States	Americas	Cross-sectional study	216	Children with AD	5-17 years	41.70	58.30	SES	Geographic location, health insurance type, family income, parent education level, and household size
Jeong and Choi [15], 2024	Korea	Asia	Survey	2048	Children with allergic conditions like atopic dermatitis, asthma, and allergic rhinitis	0 ≤ 5 years	No separate data	No separate data	Household income	Household income
Landau *et al* [16], 2024	Israel	Middle East	Cross-sectional study	77525	Children with AD diagnosis (Cases) and pediatric population attending wellness visits (Controls)	Cases: < 3 years of age, Controls: < 18 years of age	56.60	43.40	SES	Socioeconomic status
Tawfik *et al* [17], 2023	East London	Europe	Cross-sectional study	460	Infants & children, young adults of Bangladeshi origin with atopic eczema	2 months to 30 years old	57.00	43	SES	Job/income
Andersson *et al* [18], 2023	Greenland	Americas	Cross-sectional study	839	Pediatric population with AD	0–7 years	49.80	50.20	SES	Parental educational background, housing status
Kim *et al*[19], 2023	Korea	Asia	Cross-sectional study	980	Participants with the presence of allergic conditions, asthma/AD	Less than or equal to 18 years of age	64.10	35.90	SES	Household income
Agiwal *et al* [20], 2023	India	Asia	Prospective, descriptive study	380	Pediatric AD population	Up to 15 years of age	56.30	43.70	SES	Socio-economic status, residential area
Weil *et al*[21], 2022	Israel	Middle East	Retrospective case-control study	93432	Pediatric AD population	Less than 6 months to more than or equal to 18 years of age	47.70	52.30	SES	Residential area, socioeconomic status

AD: Atopic dermatitis; SES: Socioeconomic status.

**Table 2:** Primary outcome measurement variables[14-21]

Primary outcome measurement variables	Number of studies (out of 8)
Household income/income	4
Residential area/geographical location	3
Socioeconomic status	1
Parental or participant education	2
Occupation	1
Housing characteristics	1
Health Insurance	1

**Table 3:** Association between atopic dermatitis and socioeconomic status, and sub-analysis between atopic dermatitis and parental education[14-21]

Ref.	AD association with SES	Maternal education association with AD	Paternal education association with AD	Parental education association with AD
Reimer-Taschenbrecker *et al* [14], 2023	Mixed	No data	No data	Positive
Jeong and Choi[15], 2024	Positive	No data	No data	No data
Landau *et al*[16], 2024	Mixed	No data	No data	No data
Tawfik *et al*[17], 2023	No	No data	No data	Positive
Andersson *et al*[18], 2023	No data	Positive	Positive	No data
Kim *et al*[19], 2023	Mixed	Positive	Positive	No data
Agiwal *et al*[20], 2023	Mixed	No data	No data	No data
Weil *et al*[21], 2022	Mixed	No data	No data	No data

AD: Atopic dermatitis; SES: Socioeconomic status.

**Table 4:** Sub-analysis of residential area[14-21]

Residential area	Number of studies reporting data	Key findings
Urban	4	51.4% (Reimer-Taschenbrecker A), 63.4% (Landau T), 70.3% (Kim J), 58.9% (Agiwal PS)
Suburban	1	48.1% (Reimer-Taschenbrecker A)
Rural	4	0.5% (Reimer-Taschenbrecker A), 36.6% (Landau T), 29.7% (Kim J), 41.1% (Agiwal PS)
Apartment/attached house	1	82.1% (Andersson AM)
House	1	17.8% (Andersson AM)

**Table 5:** Risk of bias[14-21]

Criteria	Reimer- Taschenbrecker *et al* [14]	Jeong and Choi [15]	Landau *etal* [16]	Tawfik *etal* [17]	Andersson *etal* [18]	Kim *etal* [19]	Agiwal *etal* [20]	Weil *etal* [21]
Did the study address a clearly focused issue?	√	√	√	√	√	√	√	√
Was the cohort recruited in an acceptable way?	√	√	√	√	√	√	√	√
Was the exposure accurately measured to minimize bias?	√	√	√	√	√	√	√	√
Was the outcome accurately measured to minimize bias?	√	√	√	√	√	√	√	√
Have the authors identified all important confounding factors?	√	√	√	√	√	√	√	√
Have they taken account of confounding factors in the design/analysis?	√	√	√	√	√	√	△	√
Was the follow-up of subjects complete enough?	×	×	√	×	×	×	×	△
Was the follow-up of subjects long enough?	×	×	√	×	×	×	×	√
What are the results of this study?	√	√	√	√	√	√	√	√
How precise are the results?	√	√	√	√	√	√	√	√
Do you believe the results?	√	√	√	√	√	√	√	√
Can the results be applied to the local population?	√	√	△	△	√	√	√	√
Do the results fit with other available evidence?	△	△	√	√	√	√	√	√
What are the implications of this study for practice?	√	√	√	√	√	√	√	√
Positive/methodologically sound	√	√	√	√	√	√	√	√
Negative/relatively poor methodology	×	×	×	×	×	×	×	×
Unknowns	△	△	△	△	△	△	△	△

√: Yes; ×: No; △: Partial/unclear empty = Not reported/unknown.
